# Lessons from “Lower” Organisms: What Worms, Flies, and Zebrafish Can Teach Us about Human Energy Metabolism

**DOI:** 10.1371/journal.pgen.0030199

**Published:** 2007-11-30

**Authors:** Amnon Schlegel, Didier Y. R Stainier

**Affiliations:** Massachusetts General Hospital, United States of America

## Abstract

A pandemic of metabolic diseases (atherosclerosis, diabetes mellitus, and obesity), unleashed by multiple social and economic factors beyond the control of most individuals, threatens to diminish human life span for the first time in the modern era. Given the redundancy and inherent complexity of processes regulating the uptake, transport, catabolism, and synthesis of nutrients, magic bullets to target these diseases will be hard to find. Recent studies using the worm Caenorhabditis elegans, the fly Drosophila melanogaster, and the zebrafish Danio rerio indicate that these “lower” metazoans possess unique attributes that should help in identifying, investigating, and even validating new pharmaceutical targets for these diseases. We summarize findings in these organisms that shed light on highly conserved pathways of energy homeostasis.

## Introduction

Major shifts in human populations to urban centers, engagement in sedentary employment and leisure activities, and over-abundance of calorie-dense, processed foods have created a milieu in which ancient metabolic pathways that evolved under pressures to extract enough energy from the environment to maintain optimal reproductive and immune function and to tolerate short bouts of fasting, while avoiding excessive weight gain that would hinder escape from would-be predators, are running amuck [1]. The consequence of excess stored energy, human obesity, is poised to negate the tremendous improvements in sanitation, obstetric care, and massive vaccination that marked the developed world in the last century. For the first time in the modern era, life expectancy is expected to decrease [2]. Recognizing that it is unlikely that the social and economic pressures that generated our “toxic” lifestyle will be reversed, current research in energy metabolism focuses on mitigating the consequences of modernity. Increasingly sophisticated approaches are required to understand the critical nodes of energy homeostasis and to develop new drug targets.

**Figure 1:**
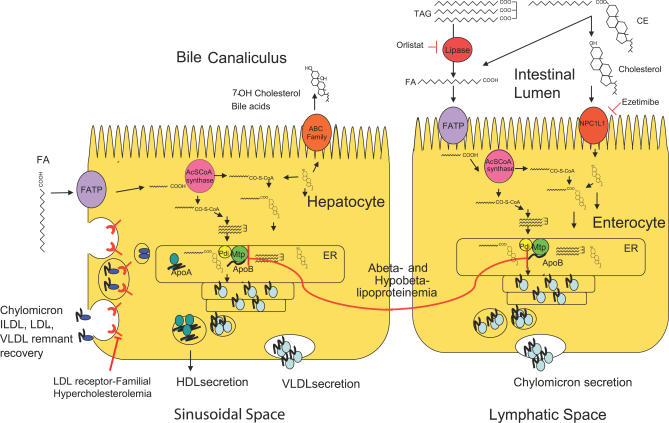
Machinery of Vertebrate Intestinal and Hepatic Lipid Transport Lipid transport in the liver and intestine involves similar cellular machinery, although the direction of traffic of nutrients and wastes is directed differentially. In the intestine, TAG, cholesterol esters (CE), and phospholipids (not shown) are hydrolyzed into FA and cholesterol by lipases (red) and cholesteryl esterases (not shown) in the intestinal lumen. FAs are transported across the apical membrane of the enterocyte by FA transport proteins (FATP, purple) and then activated with coenzyme A by acyl coenzyme A (AcSCoA) synthase (pink). AcSCoAs are used by various acyl transferases (not shown) to build TAG and CE. In the lumen of the endoplasmic reticulum (ER), microsomal triacylglycerol transfer protein (MTP) and its obligate binding partner protein disulfide isomerase (Pdi) combine neutral lipids with ApoB to make chylomicrons (blue). Chylomicrons are secreted across the basolateral membrane. Orlistat inhibits TAG hydrolysis [85] and ezetimibe blocks cholesterol uptake (red arrows) [86]. Alpha lipoproteins (high density lipoprotein particles, HDL) are also secreted by the intestine in small amounts, but the major site of synthesis is the liver. The liver retrieves free FAs from the circulation through basolateral FA transport proteins on the surface of hepatocytes; beta lipoprotein particles are retrieved by receptor mediated endocytosis on this surface as well by the low density lipoprotein particle receptor (LDL-R). Retrieved FAs are either subjected to beta oxidation in mitochondria, or reincorporation in TAG. Either cholesterol is esterified to FAs, repackaged into nascent VLDL, or subjected to hydroxylation and subsequent secretion into the bile. De novo synthesis of cholesterol and FAs also occurs in the hepatocyte, a process regulated by sterol regulatory element binding proteins and various nuclear receptors (not shown). Familial hypercholesterolemia is due to defects in LDL-R. Abetalipoproteinemia is due to mutations in MTP. Hypobetalipoproteinemia is due to mutations in Apob.

The energy-producing, storing, and transferring reactions of life are a common thermodynamic inheritance of all organisms. The large catalog of in-born errors in human metabolism includes a series of mutations ranging in phenotype from mildly hypomorphic (e.g., 75% loss in activity) to completely null (e.g., absence of activity) in the key enzymes of cellular energetics that are shared by metazoans (Figure 1). Although these mutations are rare in the general population, elucidation of the genetic and biochemical underpinnings of monoallelic diseases has greatly helped focus research in more common diseases, in general [3], and of energy metabolism, in particular. Several such disorders have comparable syndromes in “lower” metazoans (Table 1). As will be discussed below, unbiased methods have been used to identify more genes whose mutation in lower metazoans leads to phenotypes that are comparable to human syndromes of altered energy homeostasis like obesity.

**Table 1:**
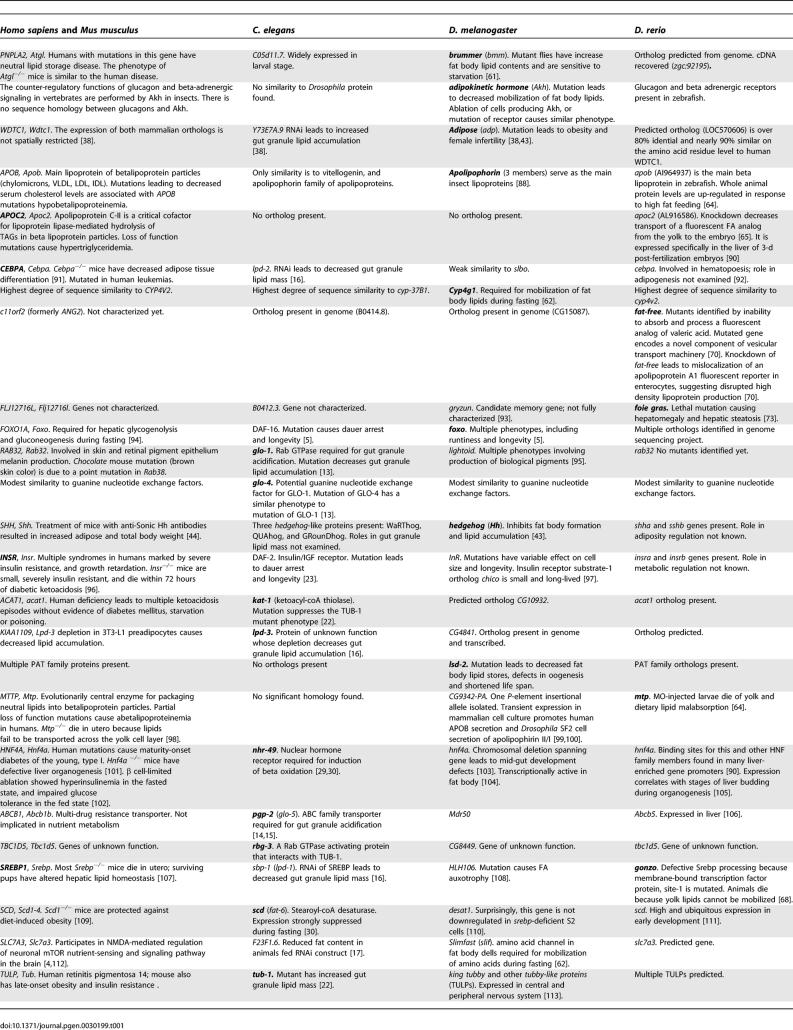
Genes Involved in Energy Metabolism Discussed in This Article (Arranged Alphabetically; Genes in Boldface Are the Focus of Discussion)

Since it is unlikely that the social and economic pressures that have generated a global pandemic of obesity will be reversed, creative tools are required to understand the signaling pathways that govern energy homeostasis and to develop new drug targets that exploit this new knowledge. In this review, we highlight metabolic research in three metazoan organisms, and argue that these studies are not mere exercises in comparative energetics (Table 2). Rather, studies on energy homeostasis in C. elegans, *Drosophila*, and zebrafish are proving that genetically tractable lower organisms can alter our understanding of the relationship of metabolic processes underlying obesity and its related illnesses (atherosclerotic vascular disease and type 2 diabetes mellitus). Through study of these organisms, insights relating energy homeostasis to life span, reproduction, and immune function have been made [4–7]. Below we focus on studies of neutral lipid homeostasis in these organisms because lipids are the main energy storage material and drug targets to combat obesity will necessarily alter their metabolism.

**Table 2:**
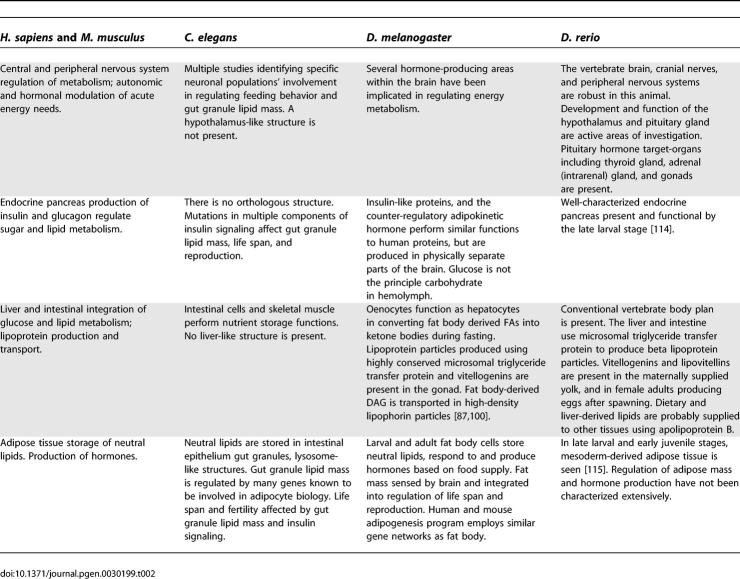
Some Parallels and Differences among Model Organisms and Human Metabolism, as Compared by Organ System

## Insulin/Insulin-Like Growth Factor Signaling, Bridging Metabolic Control and Regulation of Life Span, Reproduction, and Immune Function

The evolutionarily central insulin/insulin-like growth factor signaling pathway (IIS) has been characterized in great detail in *Drosophila* and C. elegans. Orthologous proteins for nearly all of the intermediates in this cascade are found in vertebrates. Multiple mutations in components of the IIS pathway result in alterations in life span [8], have differential effects on body size and fertility, and cause alterations in fat accumulation in their major lipid storage organs [4–7]. A consensus is emerging, however, that decreased adiposity, achieved through genetic lesions in IIS in lower metazoan, dietary restriction in rodents, and bariatric surgery in humans, can prolong healthy life span [9–11].

## C. elegans, Studying Whole-Organism Lipid Stores One Gut Granule at a Time

**Figure 2:**
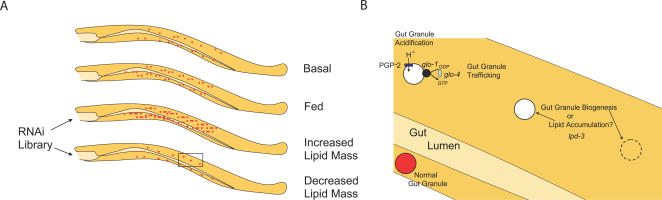
C. elegans Lipid Storage (A) C. elegans lipid stores are found in gut granules, intestinal cell lysosomes that can be visualized with polarized microscopy, and labeled for neutral lipids with fluorescent dyes like nile red (red circles). Following feeding, greater lipid mass can be appreciated by the increased number and size of lipid-laden gut granules. To identify proteins involved in regulating the amount of lipids stored in gut granules, a reverse genetic screen can be performed. Depletion of individual proteins using an RNAi library may cause increases or decreases in lipid mass. (B) Higher magnification view of the boxed region in (A) (bottom) showing the proteins involved in gut granule biogenesis and lipid accumulation. Acidification of nascent gut granules by the proton pump PGP-2 (dark blue) precedes lipid accumulation. Intracellular trafficking of nascent gut granules is governed by the GTPase encoded by *glo-1* (black) and its guanine nucleotide exchange factor, encoded by *glo-4* (light blue). The *lpd-3* gene may regulate biogenesis (dashed circle) of gut granules, be required for the accumulation of lipids nascent gut granules, or it may contribute to both processes.

In C. elegans adults, triacylglycerol (TAG) is stored in gut granules (Figure 2), enterocyte lysosomes, whose genesis, size, and traffic are amenable to genetic study [12–15]. These structures are not directly analogous to the lipid droplets seen in the *Drosophila* fat body, or in vertebrate adipose tissue; however, gut granule form and function is regulated by highly conserved nutrient sensing and intracellular signaling pathways.

Four novel genes were identified in a genetic screen for mutations causing gut granule loss (*glo* mutants) by searching for defects in the accumulation of fluorescent dyes that label acidified, mature lysosomes specifically [13]. GLO-1 is a novel Rab GTPase that associates with gut granules and is required for intestinal lysosome acidification. GLO-4 is a potential guanine nucleotide exchange factor that may activate GLO-1 by exchanging GDP for GTP (Figure 2). Whether the remaining *glo* mutants have altered neutral lipid stores, altered fertility, or changes in life span as a consequence of a defect in gut granule genesis are open questions. Likewise, the involvement of these proteins in higher metazoan neutral lipid homeostasis should be investigated.

### RNAi: Reverse genetics for system validation and comprehensive surveys.

The transcription factors C/EBP and steroid regulatory element binding protein 1 (SREBP-1) are necessary and sufficient for mammalian adipogenesis in vitro. RNAi-mediated depletion of C/EBP and SREBP-1 led to lipid depletion (*lpd* mutants) of C. elegans gut granules. [16]. Known target genes of SREBP and C/EBP, including the genes encoding enzymes of de novo fatty acid (FA) synthesis (e.g., acetyl co-A carboxylase, fatty acid synthase), were downregulated in animals depleted of these transcription factors.

A secondary screen using a library of 80 RNAi vectors known to cause larval arrest (RNAi of C/EBP or SREBP-1 causes larval arrest) uncovered eight more proteins whose knockdown caused the *lpd* phenotype. Three of these *lpd* genes encode proteins that are components of the mitochondrial respiratory chain, suggesting an obvious cause (decreased beta oxidation) for the *lpd* phenotype [16]. LPD-3 is a novel protein of unknown function whose depletion caused decreased gut granule lipid accumulation. The *lpd-3* gene is expressed in worm intestine and mammalian adipose tissue. A short-hairpin RNA strategy to knock down LPD-3 in 3T3-L1 preadipocytes caused decreased lipid accumulation, indicating the conserved function of this protein [16].

In a comprehensive RNAi survey, depletion of 305 proteins led to decreased intestinal lipid stores, while depletion of 112 proteins led to increased intestinal lipid stores [17]. Most proteins in both pools have mammalian orthologs, and the observed phenotypes were biologically plausible in most cases: many proteins known to be involved in energy homeostasis were identified. This tractable list of genes should guide not only future studies in C. elegans, but should also prioritize human and rodent investigations. For example, PGP-2 (*glo-5*), an ABC family transporter identified in this screen, was subsequently found to be required for gut granule acidification [13–15]. Like GLO-1 and GLO-4, PGP-2′s identification links gut granule biogenesis to neutral lipid storage (Figure 1). Whether mutation of PGP-2 affects transport of lipids or sterols into the nascent gut granule is unresolved, but it can be addressed by further screening for mutations that suppress the *glo* phenotype.

### What does Tubby do?

The spontaneously obese *Tubby* mouse has a mutation in a gene of unknown function. Mouse Tub is expressed in the central nervous system, and there is evidence that it is a transcription factor [18,19], an adaptor molecule involved in insulin receptor signaling [20], or a regulator of vesicular transport [21]. A worm *tub-1* mutant showed a modest decrease in gut granule lipid accumulation. Five alleles of a 3-ketoacyl-coA thiolase gene, *kat-1* were identified in a screen for mutations that could suppress the *tub-1* mutant phenotype [22]. KAT-1 may be localized to mitochondria or peroxisomes in a dynamic manner and may be required for FA oxidation. Since KAT-1 is expressed in muscles and enterocytes, it was surprising that the *tub-1* mutant phenotype was rescued through either restoring normal KAT-1 expression in muscle and intestine, or in intestine alone. Restoring KAT-1 expression in muscle alone did not affect the increased lipid mass in the intestine. Mutations causing alterations in ciliated neurogenesis recapitulated the genetic interaction of *tub-1* and *kat-1*, suggesting that a neurohormonal mechanism regulates intestinal lipid stores [22]. Finally, *tub-1* regulation of fat stores is not regulated by the insulin receptor ortholog *daf-2*, nor does it require the downstream forkhead family transcription factor *daf-16*. Both *daf-2* and *daf-16* regulate life span in addition to lipid metabolism [23,24], indicating that *tub-1* can selectively modulate lipid metabolism without impacting longevity [25].

TUB-1 interacts with a Rab GTPase activating protein RBG-3 in ciliated neurons where TUB-1 undergoes both dendritic and ciliary transport, suggesting that TUB-1′s function in the worm is to modulate chemosensory or neurohormonal functions [25]. Given the role of serotonergic neurons in regulating feeding behavior and regulating lipid stores in the worm [26], it shall be important to determine if TUB-1 modulates serotonergic neuron function, and whether the underlying signaling pathways between these neurons and the periphery communicate with those regulating longevity [27,28].

### Nutrient sensing: Coordinating a global response to fasting.

In response to a 1-h fast of adult worms, the abundance of 18 of 97 mRNAs encoding genes involved in glucose and lipid metabolism examined by reverse transcriptase polymerase chain reaction is modulated [29]. Induced “fasting response” genes include those involved in mobilization of fat stores for energy production, while some mitochondrial beta oxidation genes are repressed. Four FA desaturases are induced, while one (stearoyl-coA desaturase, SCD) is repressed strongly. This differential gene expression leads to global changes in FA composition [29]. Expression of *nhr-49*, which encodes a nuclear (hormone) receptor required for induction of beta oxidation, is also upregulated. Conversely, deletion of *nhr-49* results in elevated fat content and shortened life span. NHR-49 regulation of life span is dependent on SCD, but NHR-49 regulation of beta oxidation was independent of SCD [30]. This observation raises the possibility that SCD activity is required not for short-term catabolism, but for generation of lipid signaling molecules that have long-term effects [31]. The functions of the *Drosophila*, zebrafish, mouse, and human orthologs of NHR-49 are summarized in Table 1.

## *Drosophila*, a Fat Body for the Ages

The *Drosophila* fat body is a structure that stores maternally supplied lipids, and “feeds” the developing animal during its larval stages. As these lipid stores are consumed, most of the fat body cells die [32], although a few dissociated cells persist into adulthood, and are required to supply the animal with energy prior to the onset of feeding [33]. Adult males also require these remnant cells for proper mating behavior, suggesting that they are involved in hormone production [34]. Genetic manipulation of insulin signaling has the most dramatic effect on lipid mobilization from the fat body, with loss of insulin signaling leading to poor growth, runtiness, infertility, and longevity in certain cases [5].

Many of the associations among various aspects of physiology and *Drosophila* fat mass were made over four decades ago with the *female sterility(2)adipose* mutant (*adipose*, *adp*), a spontaneous mutant found to have increased fat body size and female infertility [35,36]. *Adp* mutants are also protected from starvation-induced death owing to this increased and “available” energy storage pool [37]. The *adp* gene encodes a WD40/tetratricopeptide-repeat-domain protein [38] whose function in regulating TAG accumulation is conserved in worms and mice as assessed through RNAi in the former, and by exhaustive transgenesis and knockout strategies in the latter [39]. The Adp protein regulates TAG accumulation in fat body cells by modulating chromatin acetylation and modulating PPARγ activity [39]. In light of this definitive study, the human ortholog *WDTC1* is a candidate gene for obesity worth pursuing.

Adipocytes are mesodermal cells generated by well characterized transcriptional networks [40,41]. The Hedgehog (Hh) morphogens, a group of cholesterol-modified proteins involved in multiple developmental processes [42], inhibit *Drosophila* fat body development and lipid accumulation [43]. Treatment of mice with neutralizing anti-Sonic Hh antibodies resulted in increases in adipose and total body weight [44]. Specific inhibition of Hh signaling in adult mesodermal stem or progenitor cells might be useful treatments for lipodystrophies, rare acquired and inherited diseases marked by adipose tissue loss and TAG redistribution [45]. Conversely, specific activation of Hh signaling could be useful for treatment of obesity.

### Fat body regulation by an insulin signaling network.

**Figure 3:**
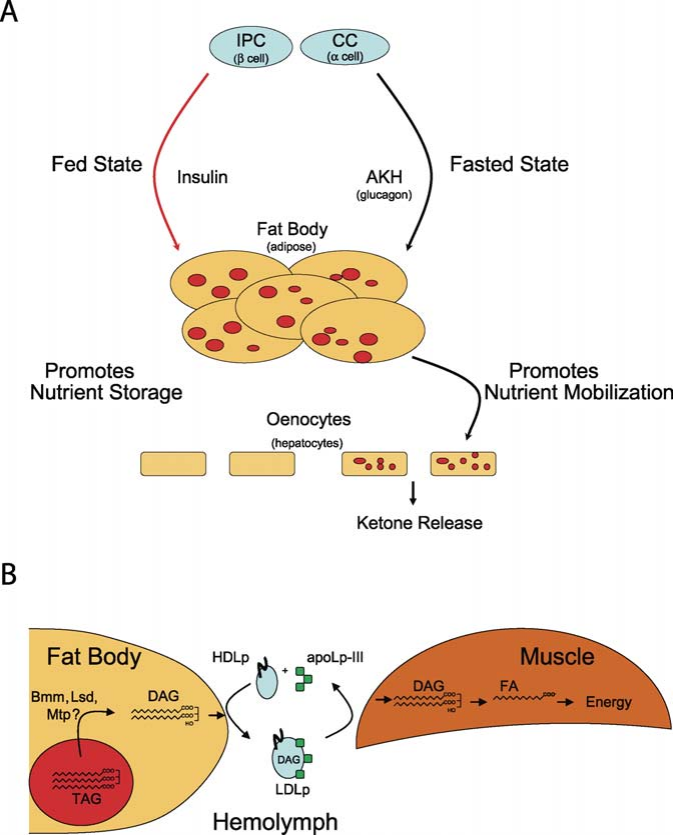
*Drosophila* Metabolic Integration and Lipoprotein Transport In the fed state, IPCs release insulin. In the fat body, insulin promotes nutrient storage. In the fasted state, AKH is released from the CC cells. AKH promotes release of FAs from the fat body. FAs are retrieved by oenocytes in the fasted state where they are stored as triacylglycerol in lipid droplets (red circles) and metabolized to generate ketone bodies. These metabolites are then released into the circulation for use as fuel until the next feeding. The parallel cells and tissues in vertebrates are shown in parentheses, although their physical arrangement is different (e.g., vertebrate α and β cells reside in pancreatic islet of Langerhans). Also, AKH and glucagon show no structural similarity, despite their related function. (B) During flight, *Drosophila* neutral lipid stored as TAG is partially hydrolyzed into DAG in a process regulated by adipokinetic hormone (not shown). This lipid mobilization may involve lipid storage droplet proteins (LSD), the Brummer lipase (Bmm), and microsomal triglyceride transfer protein (Mtp). Liberated DAGs combine with high density lipophorin particles (HDLp), which have the coat proteins apolipophorins I and II (stylized as a black curve on the surface of the blue sphere), to make low density lipophorin particles (LDLp) when combined with apolipophorin III (green). This LDLp particle delivers DAGs to active skeletal muscles, which extract DAG from LDLp. Myocytes hydrolyze the delivered DAG molecules into FAs. Beta oxidation generates the energy required to sustain flight [87]. Also, lipids are delivered to insect fat body cells by HDLp, which is bound to an insect lipophorin receptor (iLR) that is internalized [88], but unlike endocytosed vertebrate LDL, insect HDLp is not degraded. Rather, HDLp is recycled to the hemolymph after lipids are extracted from the lipoprotein particle [89].

*Drosophila* has cell types reminiscent of vertebrate metabolic organs that regulate fat body lipid stores (Table 2): endocrine pancreas-like cells and hepatocyte-like cells are found in *Drosophila*. Their contributions to regulating adipose and integration into other aspects of life are being appreciated (Figure 3). Ablation of the insulin producing cells (IPCs) in the brain of *Drosophila* causes developmental delay, runtiness, elevated carbohydrate levels in larval hemolymph, and increase lipid stores [46,47].

In insects, insulin function is “counter-regulated” by adipokinetic hormone (AKH), a neuropeptide that functions like glucagon and β-adrenergic agonists do in vertebrates in counteracting insulin action. AKH is particularly important for mobilizing energy stores for active flight muscles [48,49], when lipid droplet TAG stores are partially hydrolyzed into diacylglycerol (DAG) and incorporated into lipoprotein particles that deliver fuel to active muscles (Figure 3). AKH is produced by the corpora cardia (CC) cells in another part of the brain called the ring gland that is physically separate from the IPCs [50]. AKH secretion is under control of the same ATP-sensing apparatus used by vertebrate pancreatic β cells to control insulin release [51], suggesting conservation of function, if not form, among animals.

### Lipid droplet dynamics.

Study of lipid droplets in *Drosophila* fat body cells have shown that the proteins coating these storage structures are similar to those in mouse adipose tissue in form and function [52]. The *Drosophila* PAT (Perilipin, Adipophilin, and Tip47) family lipid droplet coat protein lipid storage droplet-2 (Lsd-2) is expressed in the ovary and the fat body [53,54]. Mutation of *lsd-2* results in defects in oogenesis [54], decreased fat body TAG accumulation, and shortened life span [53]. These defects in lipid storage and handling reflect perturbations in lipid droplet motion within cells expressing mutated Lsd-2 [55]. Dynamic phosphorylation of Lsd-2 [55] and multiple other proteins in the lipid droplet sub-proteome occurs during intracellular trafficking of these highly mobile energy storage depots [56].

In response to AKH, fat body TAG lipase is activated, and multiple lipid droplet proteins, Lsd-2 chief among them, show increased phosphorylation. Furthermore, lipid droplet protein phosphorylation in response to AKH requires cyclic AMP and protein kinase A, reminiscent of β-adrenergic stimulation of mammalian lipolysis [57]. Conversely, mutation of the adipokinetic hormone receptor gene (*AKHR*), or ablation of AKH-producing CC cells leads to increased fat body TAG stores, and to an inability to release FAs from depot in response to starvation. This defect in fat body lipolysis in animals lacking AKH signaling is manifested by their earlier death when food is withdrawn [58].

The similarity between mammalian and *Drosophila* TAG mobilization is underscored by observations involving the *brummer* (*bmm*) mutant fly and human neutral lipid storage disease: both are caused by mutations in the gene encoding adipocyte TAG lipase (ATGL, PNPLA2) [58,59]. Humans with mutated ATGL experience neutral lipid droplet accumulation in many organs, leading to widespread impairment of normal function. Similarly, *Atgl* ^−*/*−^ mice have massive intracellular liposis in many tissues, including the heart [60]. In *bmm* mutant flies, increased TAG stores, frank obesity, and an increased sensitivity to starvation (i.e., earlier death) are seen. This final phenotype contrasts with that seen when *adp* mutant flies are fasted, indicating that expanded lipid mass does not necessarily mean increased availability of calories during fasting [38]. The *bmm* mutant phenotype can be antagonized by introducing an *lsd-2* loss of function mutation; *bmm*/*lsd2* double mutants have normalized TAG stores [61]. Mobilization of fat body TAG stores is under hormonal regulation and may require lipases other than Bmm: *AKHR/bmm* double mutants have higher fat body TAG stores than either *AKHR* or *bmm* individual mutants, and are more sensitive to starvation than either mutant [58].

### The oenocyte: A proto-hepatocyte.

During fasting, mobilized adipose TAG stores are sent to the liver as free FAs for partial oxidation to ketone bodies, molecules that can be used by nearly all cell types as energy sources (Figure 3). The *Drosophila* oenocyte functions in the capacity of ketogenic hepatocyte during a fasting response [62]. Whereas eonocytes show little lipid stores in the fed state, knockdown of the Slif amino acid channel in fat body cells, a manipulation that prompts protein catabolism and mobilization of amino acids for ketogenesis in the “liver,” results in oenocyte lipid accumulation. Similarly, knockdown of fat body cell *lsd-2* decreases oenocyte lipid staining during starvation. Conversely, overexpression of Brummer lipase in fat body cells promotes oenocyte lipid accumulation during fasting. Taken together, these findings suggest that oenocyte lipid stores are derived from fat body cells, much like vertebrate hepatocytes' lipids are derived from TAGs that are mobilized from adipocytes during fasting. Finally, targeted ablation of oenocytes leads to runtiness and failure to mobilize fat body lipid stores during fasting, a process that requires the lipid ω-hydroxylase Cyp4g1 [62].

## Zebrafish: Vertebrate Metabolism Comes into Genetic Focus

**Figure 4:**
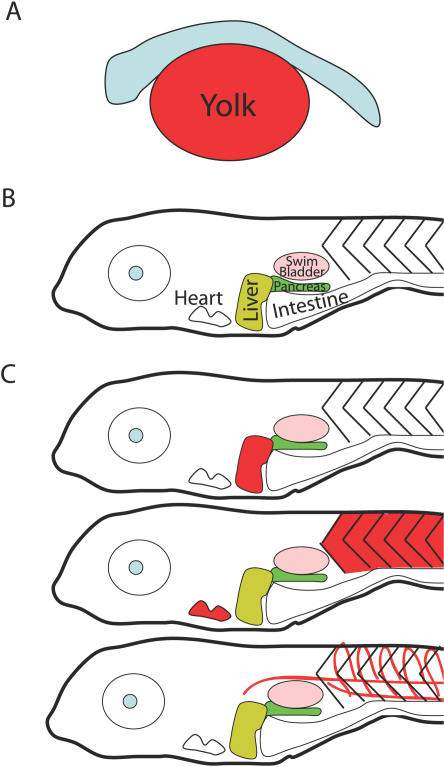
Zebrafish Larval Anatomy and Detection of Mutations in Lipid Homeostasis (A) Zebrafish embryos and larvae subsist on yolk lipids for the first 4 d of life. (B) By 7 d post-fertilization, only the surfactant-filled swim bladder (lavender) stains faintly with Oil Red O. The liver, pancreas (green), intestine, and heart are visible through the skin at this stage of development. The skeletal muscles (chevron-shaped somites) occupy the posterior of the animal. (C) Mutations causing inappropriate lipid accumulation in the liver (upper), skeletal and cardiac muscles (middle), and vasculature (bottom) can be easily visualized.

As a vertebrate, zebrafish possesses many structural similarities with humans that *Drosophila* and C. elegans do not (Table 2). For instance, zebrafish digestive organs, adipose tissues, and skeletal muscle are physically arranged in a manner similar to their human counterparts (Figure 4). Since zebrafish eggs are fertilized externally, the development and onset of their metabolic activity can be monitored in real time in live organisms.

### Probing the machinery of lipoprotein transport.

The machinery of lipid synthesis and transport used by humans is present and active in zebrafish (Figure 1). Beta lipoproteins are central to the trafficking of yolk-supplied, intestinal, and hepatic lipids. Like *Drosophila*, zebrafish have several members of this family, including the yolk lipoproteins (e.g., vitellogenin), and the hepatic and intestinal protein apolipoprotein B (Apob). The packaging of lipids into beta lipoprotein particles is the task of the evolutionary progenitor of this family of proteins, microsomal triglyceride transfer protein (Mtp), which is also present in the zebrafish yolk cell layer, intestine, and liver [63]. Knockdown of zebrafish Mtp expression using anti-sense morpholino oligonucleotides (MOs) results in many of the phenotypes seen in severe, lethal human abetalipoproteinemia and in *Mtp* ^−*/*−^ mice, with failure to transport yolk lipids and die in utero by starvation [64].

While knockdown of zebrafish Mtp recapitulates many of the phenotypes of severe, lethal abetalipoproteinemia seen in *Mtp* ^−*/*−^ mice, including death by failure to transport neutral lipids across the yolk cell layer, an intermediate phenotype of decreased, but not absent, vascular lipid staining was not seen in Mtp MO-injected zebrafish larvae. This “intermediate” phenotype is present in *Mtp^+/^* ^−^ mice and many humans with point mutations in the orthologous *MTTP* gene [64]. This distinction is important because there is heterogeneity in the clinical phenotype of abetalipoproteinemia, ranging from “silent” low levels of serum cholesterol to severe malabsorption of dietary lipids and fat-soluble vitamins. Furthermore, whole organismal Apob levels were not affected by Mtp knockdown, but we could not assess serum Apob levels. These limitations indicate that advances in methodology and flexibility in using complementary systems to follow up on findings in zebrafish lipid metabolism will be needed. Likewise, care must be taken in interpreting studies that rely solely on targeted knockdown of proteins in this model system.

Unlike C. elegans, which has comprehensive RNAi libraries for transient knockdown of most genes, a large pool of validated MOs is not available for systematic knockdown of zebrafish genes. One study illustrates this limitation. Following a proteomic survey to identify cotranslationally translocated (membrane and secretory) proteins in zebrafish embryos, a tractable set (150) of the corresponding genes was selected for targeted knockdown with antisense MOs [65]. Knockdown of only one of these proteins, apolipoprotein C2 (Apoc2), affected transport of a fluorescently tagged 12-carbon FA injected into the yolk. Whether other MOs used in this study simply failed to decrease protein expression is unclear, but a likely reason for this finding is that the maternal supply of the complement of cotranslationally translocated proteins required for yolk lipid transport may be sufficiently large that knockdown of any one component will not affect lipid transport.

Analogous redundancy and compensation for loss of one of three Δ9 FA desaturases occurs in worms [66], where individual mutations of the corresponding genes leads to minimal phenotypic difference. When the three possible pairs of Δ9 FA desaturase mutants are examined, differential effects on lipid stores are apparent, indicating subtle differences in function [67]. Adding to the complexity of this metabolic pathway, the expression of the three Δ9 FA desaturases is differentially regulated by NHR-49 [30], raising the tempting possibility that these enzymes have unique substrates and differ in their capacities to generate lipid signaling molecules. As more robust MO libraries are generated, targeted knockdown in zebrafish may approach this level of genetic sophistication.

The *gonzo* mutant also demonstrates the high degree of conservation of metabolic regulatory function across animal phyla: the affected gene encodes the site 1 protease responsible for processing the ER membrane-tethered transcription factor SREBP, the master regulator of de novo cholesterol and FA synthesis [68]. *gonzo* mutants are incapable of responding to low cellular cholesterol by activating SREBP, and are thus genetically “starved” of lipids. This defect is manifested by an inability to mobilize the yolk lipid stores, a phenotype that is readily apparent with conventional staining methods.

In addition to confirming the conservation of known genes in regulating lipid metabolism, zebrafish has been useful in identifying novel proteins involved in energy homeostasis. The *fat-free* mutant fails to concentrate a fluorescent FA analog in its gall bladder [69]; the affected gene encodes a novel component of the vesicular transport machinery [70]. While *fat-free* mutants appeared morphologically normal (and would have been missed in the first, large scale screens for intestinal morphology), they have multiple defects in intracellular transport. Since a large-scale genetic screen in medaka has found three more mutants with defective hepatobiliary processing of the same fluorescent substrate used to identify *fat-free*, further insights into these processes are expected from genetic studies with fish [71].

### Non-alcoholic fatty liver disease: Modeling complex human metabolic diseases.

One obesity-related disease receiving increasing attention is non-alcoholic fatty liver disease (NAFLD). The initial step in the pathogenesis of NAFLD is the accumulation of excess neutral lipid in the liver, a condition commonly seen in overweight and insulin-resistant persons [72]. Two mutations from a library of mutations in essential genes show hepatic steatosis [73]. Whether these mutations are due to excessive de novo hepatic lipid production, decreased hepatic secretion of very low density lipoprotein particles, diminished beta oxidation of FAs in the liver, more subtle defects in regulating energy homeostasis including insulin resistance, or some non-cell-autonomous cause is unknown. We await future studies of these two genes, particularly of *foie gras*, whose encoded protein exhibits no obvious domains and represents a completely novel pathway to inappropriate accumulation of lipids in the liver.

## Conclusions

To address the looming public health crisis of obesity and its related illnesses, increasing attention is being turned to the three work-horse organisms of modern genetics outlined in this review. Future work with these organisms must exploit their individual strengths and avoid their limitations (Table 2). In this section we outline the advances in metabolic research we expect to be made in the near term in each organism.

In C. elegans, integration of acute and long-term signals among the nervous system, skeletal muscles, and intestine should emerge from studies of gut granule biogenesis, ciliated neurogenesis, and feeding behavior. These studies will parallel and inform discoveries into the mechanisms by which life span and fertility are regulated.

The accelerated pace of advances in studying the molecular genetics of energy homeostasis in fruit flies should allow investigators to ask increasingly sophisticated questions about physiology. In particular, understanding the signals emanating from the fat body to the IPCs, CC cells, and oenocytes should be informative. Further genetic dissection of the regulation of Brummer lipase, particularly through interaction with PAT proteins, should tell us more about how lipid droplets are regulated. Finally, through exploration of the form and function of the proto-pancreatic and hepatic organs, we can expect new insights into the integration of energy homeostasis and other facets of life including reproduction, life span, and immune function.

Future screens of energy metabolism in zebrafish must harness the optical transparency and power of transgenesis to label individual cells with fluorescent protein to monitor real-time changes in energy state [74]. Examination of the effects of targeted cell ablation of pancreatic β cells should be informative [75]. Mesodermal differentiation into adipose tissue is also an exciting area amenable to study in zebrafish [76]. More generally, improvements in reverse genetic strategies, including robotic systems for injection of antisense MOs [77], and photoactivation of knock-down reagents to allow for spatial and temporal control of knockdown, should be adopted [78,79].

Broader questions of central nervous system regulation of energy homeostasis could also be addressed in zebrafish. Vertebrate hypothalamic [80] and pituitary development [81] are active areas of investigation, and further refinements in our understanding of how these structures are formed can be expected. Genetic screens to identify mutations modulating the activity of the neurohormonal circuits governing adipose mass, the “adipostat,” are awaited also [82].

Ultimately, technical advances and more detailed characterization of the known pathways regulating lower metazoan energy metabolism should aid in identifying and characterizing novel candidate genes for human diseases such as obesity, diabetes mellitus, lipodystrophy, and non-alcoholic fatty liver disease. Conversely, the function of completely novel genes implicated in obesity [83] and diabetes [84] may be amenable to more streamlined characterization in lower organisms. The limitations of each model system (Table 2) should be recognized in advance when deploying these organisms for target validation. However, the ability to perform ever-more detailed and mechanistic studies indicates that such an approach should be productive.

### Accession Numbers

The National Center for Biotechnology Information (NCBI) Entrez database (http://www.ncbi.nlm.nih.gov/sites/gquery) accession numbers for the genes, gene products, and conditions discussed in this paper are: *ACAT1*/*acat1*, OMIM: *607809; *adp*, FBgn0000057; AKH, FBgn0004552; *Apob*, OMIM: +107730; *Apoc2*, AL916586/OMIM: *608083; *ATGL/PNPLA2*, OMIM: *609059; *B0412.3*, WBGene00015173; *B0414.8*, WBGene00015176; C/EBP, OMIM: *116897; *c11orf2*/formerly *ANG2*, *601922; *CG10932*, FBgn0029969; *CG15087*, FBgn0034380; *CG4841*, FBgn0032622; *CG8449*, FBgn0038129; *CG9342*, FBgn0032904; *chico*, FBgn0024248; *C05d11.7*,WBGene00015484; *cyp-37B1*, WBGene00009226; *Cyp4g1*, FBgn0010019; *CYP4V2*, OMIM: *608614; *daf-16*, WBGene00000912; *daf-2*, WBGene00000898; *desat1*, FBgn0086687; *F23F1.6*, WBGene00017747; *fat-free*, NP_001036200; *foie gras,* ENSDARG00000004726; *foxo*, FBgn0038197; *FOXO/Foxo* OMIM: *136533; GLO-1, NP_001024837; *glo-1*,WBGene00011083; GLO-4, WBGene00017195; *gonzo*, NP_954683; *gryzun*, FBgn0035416; Hh, FBgn0004644; *HLH106*, FBgn0015234; *HNF4A*/*Hnf4a*, OMIM: *600281; *InR*, FBgn0013984; *INSR*/*Insr*, OMIM: *147670; *kat-1*, WBGene00002183; *king tubby*, FBgn0015721; *lightoid*, FBgn0002567; LOC570606, XP_699199; *lpd-2*, WBGene00003059; *LPD-3*, WBGene00003060; *Lsd-2*, FBgn0030608; *Mdr50*, FBgn0010241; *Mtp*, OMIM: #200100; *nhr-49*, WBGene00003639; PGP-2/*glo-5*, WBGene00003996; *Rab32*, OMIM: *606281; RBG-3, WBGene00007167; *sbp-1*/*lpd-1*, WBGene00004735; *scd*/*fat-6*, WBGene00001398; *SCD*/*Scd1–4*, OMIM: *604031; *slbo*, FBgn0005638; *SLC7A3*/*Slc7a3*, OMIM: *300443; *slif*, FBgn0037203; *SREBP1*/*SREBF1*, OMIM: *184756; Tub, OMIM: *601197; *tub-1*, WBGene00006655; and *zgc:92195*, NP_001002338.

## Abbreviations

*adp*: adipose mutant
AKH: adipokinetic hormone
Apob: apolipoprotein B
ATGL: adipocyte triacylglycerol lipase
CC: corpora cardia
DAG: diacylglycerol
FA: fatty acid
*glo*: gut granule loss mutant
HDLp: high density lipophorin particles
Hh: hedgehog
IIS: insulin/insulin-like growth factor signaling
iLR: insect lipophorin receptor
IPC: insulin producing cell
kat-1: 3-ketoacyl-coA thiolase 1
LDLp: low density lipophorin particles
*lpd,*: lipid-depleted mutant
Lsd: lipid storage droplet protein
MO: morpholino oligonucleotide
Mtp: microsomal triglyceride transfer protein
NAFLD: non-alcoholic fatty liver disease
PAT: perilipin, adipophilin, and Tip47
RNAi: ribonucleic acid interference
SCD: stearoyl-coA desaturase
SREBP: sterol regulatory element binding protein
TAG: triacylglycerol
